# Validation of a Cox prognostic model for tooth autotransplantation

**DOI:** 10.1002/cre2.819

**Published:** 2023-11-28

**Authors:** Toshiya Yoshino, Michiko Yoshizawa, Shoko Aoyama, Toshiko Sugai‐Toyama, Kanae Niimi, Nobutaka Kitamura, Tadaharu Kobayashi

**Affiliations:** ^1^ Division of Reconstructive Surgery for Oral and Maxillofacial Region, Department of Tissue Regeneration and Reconstruction Niigata University Graduate School of Medical and Dental Sciences Niigata Japan; ^2^ Department of Oral and Maxillofacial Surgery, School of Dentistry Matsumoto Dental University Nagano Japan; ^3^ Patient Support Center Niigata University Medical and Dental Hospital Niigata Japan; ^4^ Protocol Data Center Niigata University Medical and Dental Hospital Niigata Japan

**Keywords:** Cox prognostic model, external validation, tooth autotransplantation

## Abstract

**Objectives:**

This study aimed to validate our Cox proportional hazards prognostic model for autotransplantation of teeth with complete root formation using prognostic index (PI) and determine whether the prognosis can be predicted.

**Patients and Methods:**

The Protocol group, as a training data set for validation, consisted of 259 autotransplanted teeth to create a PI using the Cox model, as described previously. The Pre‐protocol group, as the first validation data set, consisted of 95 autotransplanted teeth treated without a protocol. The Post‐protocol group, as the second validation data set, consisted of 61 autotransplanted teeth obtained after the establishment of the prognostic model. Because four prognostic factors, including history of root canal treatment (yes), number of roots (multirooted), source of donor tooth (maxillary tooth), and duration of edentulism (≥2.5 months), were selected as a Cox prognostic model, 16 patterns of PI were constructed. First, the autotransplantated teeth in the Protocol group were divided into low‐ and high‐risk groups respectively according to the median of PI as the cutoff value. The survival curves of low‐ and high‐risk groups were calculated using the Kaplan–Meier method and tested using the log‐rank test. Then, in the Pre‐ and Post‐protocol groups, all transplanted teeth were divided into low‐and high‐risk teeth by the median of PI and the survival curves of low‐ and high‐ risk teeth were analyzed statistically in a similar manner.

**Results:**

The survival curves of the low‐ and high‐risk groups diverged significantly in the Protocol and Post‐protocol groups. In the Pre‐protocol group, the curves of the low‐ and high‐risk groups were separated, and the low‐risk survival rate was improved.

**Conclusions:**

Our Cox prognostic model for autotransplantation of teeth with complete root formation was useful in predicting the prognosis by external validation using PI.

## 1 INTRODUCTION

Autotransplantation of teeth with complete root formation has been indicated for the replacement of one or more teeth that are lost because of dental caries or periodontitis in adults (Bae et al., 2010; Kim et al., 2005; Kvint et al., 2010; Mejàre et al., 2004). Transplanted teeth are influenced by pre and perioperative conditions (Schwartz et al., 1985). The clinical outcomes and influence of prognostic factors for autotransplantation of teeth with incomplete and complete root formation have been statistically analyzed in numerous studies. Andreasen et al., performed a long‐term study of 370 autotransplanted premolars and clarified the prognosis and risk factors of the transplantation of developing teeth (Andreasen et al., 1990; Andreasen et al., 1990; Andreasen et al., 1990; Andreasen et al., 1990). Schwartz et al. retrospectively analyzed 291 autotransplanted teeth with incomplete and complete root formation and analyzed the prognostic relevance of clinical and radiological factors (Schwartz et al., 1985). Our group had performed prospective studies to statistically evaluate the clinical outcomes and influence of prognostic factors for autotransplanted teeth with complete root formation and advocated a prognostic model using the multivariate Cox proportional hazards regression analysis (Aoyama et al., 2012; Sugai et al., 2010). A history of root canal treatment, number of roots, source of donor tooth, and duration of tooth absence were found to be statistically associated with unsuccessful transplantation of teeth with complete root formation (Aoyama et al., 2012). However, in general, a prognostic model should not be used in clinical practice unless it has been demonstrated to have a beneficial role and can help discriminate between positive and negative outcomes in patients not included in the model development (Moons et al., 2009; Royston & Altman, 2013). In our series of studies, the contribution of our model in predicting the prognosis of autotransplanted teeth with complete root formation remains unclear. Therefore, evaluating the validity and the reliability of this model for prognostic predictions is necessary. The Cox prognostic model is considered as a model consisting of its regression coefficients and their covariance matrix. The application of the model requires the prognostic index (PI) and the baseline survival function (Royston & Altman, 2013). We applied the external validation using PI proposed by Royston & Altman (2013) to validate our model.

To analyze the validity of our prognostic model, the data set used in our previous study, which advocated a prognostic model (Aoyama et al., 2012), was defined as the training data set (or “derivation data set”). The patients in this data set were treated using a specific protocol to enable objective evaluation of data concerning patient information, treatment, and outcomes of autotransplanted teeth. This training data set was termed the Protocol group. In addition, we established two validation datasets: one that included patients treated without using a specific protocol (the Pre‐protocol group) and a second that included those treated using the protocol which was reassessed after our first study of prognostic factor (Sugai et al., 2010) was published (the Post‐protocol group).

The aim of this study was to validate our Cox proportional hazards prognostic model for autotransplantation of teeth with complete root formation using two validation datasets. This is the first study to validate the Cox prognostic model for autotransplanted teeth, although such studies have been widely used in oncology and other medical fields (Royston & Altman, 2013; Bleeker et al., 2003; Haroon et al., 2015; Katsuya et al., 2012; Pflug et al., 2014).

## 2 PATIENTS AND METHODS

### 2.1 Patients

**Figure A1:**
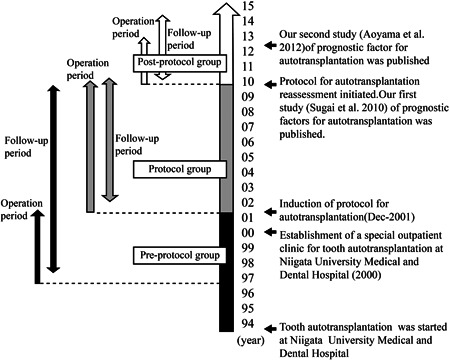
Autotransplantation of teeth during the periods of transition to a specific protocol at Niigata University Medical and Dental Hospital. The solid black, gray, and white arrows indicate the years of tooth autotransplantation in the Pre‐protocol, Protocol, and Post‐protocol groups, respectively. The Protocol group covered the longest period. The solid black, gray, and white double‐headed arrows denote the follow‐up periods for the same groups, with the follow‐up in the Pre‐protocol group being the longest.

The Protocol group, as a training derivation data set for validation of a PI with a Cox proportional hazard model, as described previously, consisted of 259 teeth with complete root formation that were autotransplanted between November 1, 2001, and March 31, 2010, at Niigata University Medical and Dental Hospital, with the termination of follow‐up by June 30, 2010 (Aoyama et al., 2012). The Pre‐protocol group, as the first validation data set, consisted of 95 teeth autotransplanted without a protocol between March 1, 1997, and October 31, 2001, before the establishment of a special outpatient clinic for tooth transplantation at Niigata University Medical and Dental Hospital; the follow‐up was terminated on March 31, 2010. The Post‐protocol group, as the second validation data set, consisted of 61 teeth autotransplanted, treated using the protocol which was reassessed after our first study of prognostic factors (Sugai et al., 2010) was published (between April 1, 2010, and March 21, 2013). It included the most recent autotransplanted teeth among the three groups; the follow‐up was terminated on September 30, 2014 (Appendix Figure A1).

Informed consent was obtained from all the patients. The study protocol was approved by the Institutional Review Board of Niigata University and conformed to the guidelines of the Declaration of Helsinki for clinical research involving human subjects (No. 2019‐0048, date of approval: March 3, 2020).

The three datasets were obtained from observational cohort studies. The Strengthening the Reporting of Observational Studies in Epidemiology guidelines were adopted in this study (Vandenbroucke et al., 2007).

### 2.2 Treatment procedures

The number of oral surgeons who treated the patients was 24, 26, and 7 in the Protocol, Pre‐protocol, and Post‐protocol groups, respectively. The treatment protocol for autotransplantation of teeth followed at the Niigata University Medical and Dental Hospital has been described previously (Aoyama et al., 2012; Sugai et al., 2010). Standardized surgical techniques were performed according to Andreasen et al., (1990). Briefly, the donor tooth was carefully extracted and positioned in a newly prepared socket. The flaps were closely sutured using 4‐0 silk sutures around the transplanted teeth. Subsequently, the tooth was splinted with orthodontic wire and resin or with sutures. The sutures were removed 7 days after surgery, and the wire splint was removed 3 weeks after surgery. Endodontic treatment was initiated 3 weeks after the surgery by an endodontist, and prosthetic treatment was completed by dentists who had recommended tooth transplantation to their patients or by dentists who worked in our hospital. The patients were examined clinically and radiographically and reviewed at 1, 2, and 3 weeks and 3, 6, 9, and 12 months after surgery according to the protocol. After 1 year, the patients were followed‐up at intervals of 6–12 months.

## 3 CORRELATION MATRIX FOR FACTORS IN EACH OF THREE GROUPS AND COMPARISON AMONG THE THREE GROUPS

The method for binarizing variable factors in the protocol was the same as that used in our previous study (Aoyama et al., 2012) (Table 1). To examine the epidemiological characteristics of tooth autotransplantation cases, a correlation coefficient matrix was used to calculate the correlation between prognostic factors in each group and subsequently compared among the three groups. The odds ratios and two‐sided 95% confidence intervals were calculated using 2 × 2 tables to estimate the relationships between the binary factors.

**Table 1:** Patient characteristics.

			Protocol group (training data set)		Pre‐protocol group (first validation data set)		Post‐protocol group (second Validation data set)	
			No.	%	No.	%	No.	%
Variable factors in protocol			259		95		61	
Patient	Age	<40 years old	124	47.9	62	65.3	33	54.1
		≧40 years old	135	52.1	33	34.7	28	45.9
	Gender	Female	104	40.2	66	69.5	38	62.3
		Male	155	59.8	29	30.5	23	37.7
	Smoking habit	No	88	34.0	92	96.8	50	82.0
		Yes	36	13.9	3	3.2	11	18.0
Donor tooth	Source	Maxillar	112	43.2	41	43.2	26	42.6
		Mandibular	147	56.8	54	56.8	35	57.4
	Type	Others	39	15.1	22	23.2	7	11.5
		Molars	220	84.9	73	76.8	54	88.5
	State of eruption	Erupted	227	87.6	85	89.5	52	85.2
		Not erupted	32	12.4	10	10.5	9	14.8
	Probing pocket depth	<4 mm	188	72.6	86	90.5	55	90.2
		≧4 mm	71	27.4	9	9.5	6	9.8
	History of dental caries and/or restoration	No	124	47.9	55	57.9	35	57.4
		Yes	135	52.1	40	42.1	26	42.6
	History of root canal treatment	No	247	95.4	89	93.7	60	98.4
		Yes	12	4.6	6	6.3	1	1.6
	Number of roots	Single‐rooted	151	58.3	52	54.7	41	67.2
		Multirooted	108	41.7	43	45.3	20	32.8
	Hypertrophy and/or curve of root	No	47	18.1	30	31.6	11	18.0
		Yes	212	81.9	65	68.4	50	82.0
	Divergence of root	No	196	75.7	76	80.0	50	82.0
		Yes	63	24.3	19	20.0	11	18.0
	Root fracture at removal	No	241	93.1	90	94.7	60	98.4
		Yes	18	6.9	5	5.3	1	1.6
Recipient site	Position	Maxilla	57	22.0	23	24.2	15	24.6
		Mandible	202	78.0	72	75.8	46	75.4
	Site	Others	37	14.3	18	18.9	9	14.8
		Molars	222	85.7	77	81.1	52	85.2
	Duration of tooth absence	<2.5 months	125	48.3	50	52.6	42	68.9
		≧2.5months	134	51.7	45	47.4	19	31.1
	Adjacent tooth	No	39	15.1	26	27.4	17	27.9
		Yes	220	84.9	69	72.6	44	72.1
Positional relation of donor tooth and recipient site	Adjacent position	No	207	79.9	75	78.9	43	70.5
		Yes	52	20.1	20	21.1	18	29.5
	Same quadrant	No	163	62.9	62	65.3	35	57.4
		Yes	96	37.1	33	34.7	26	42.6
	Opposite side	No	164	63.3	61	64.2	44	72.1
		Yes	95	36.7	34	35.8	17	27.9
	Opposite jaw	No	152	58.7	47	49.5	36	59.0
		Yes	107	41.3	48	50.5	25	41.0
Others	Method of the fixation	Sutures	53	20.5	1	1.1	4	6.6
		Wire and resin	206	79.5	94	98.9	57	93.4

### 3.1 Evaluation of prognosis

All autotransplanted teeth were evaluated as described previously (Aoyama et al., 2012). Autotransplanted teeth were classified into two groups, unsuccessful and successful, according to the postoperative results. Successful cases included transplanted teeth that healed well at the recipient site or those with only minor problems. The attending oral surgeons determined the need for extraction of transplanted teeth that were deemed unsuccessful based on the clinical findings, such as severe mobility and/or severe inflammation of the periodontal tissue. This criterion was used because a transplanted tooth was not extracted in one patient at the patient's request or for scheduling reasons, even with existing severe problems, cannot be evaluated as successful. The causes for unsuccessful outcomes were defined in the study as follows: failure of peritransplant tissue healing and root resorption.

### 3.2 Overall life table

The term “event” was defined as the judgment of transplanted teeth as unsuccessful in our series of studies (Aoyama et al., 2012; Sugai et al., 2010). The survival time was censored when there was a follow‐up time; however, an event had not yet occurred or was not known to have occurred in the survival analysis. Therefore, transplants were considered censored at the time of the last clinical and radiographical follow‐up when the transplant was registered as successful for statistical analysis (Aoyama et al., 2012; Sugai et al., 2010). To investigate the changes in the outcomes of tooth autotransplantation in the three groups, the overall life tables were calculated using the Kaplan–Meier method. The overall 1‐year rates for the three groups and the overall 5‐year rates for the Protocol and Pre‐protocol groups were calculated. In contrast, the overall 4‐year rate for the Post‐protocol group was calculated because the observation period in the Post‐protocol group was shorter than that in the other two groups.

### 3.3 Validation using the PI

In our previous study regarding the prognostic factors of tooth autotransplantation, four variables (history of root canal treatment, number of roots, source of donor tooth, and duration of tooth absence) were selected as a Cox prognostic model. The *β* coefficients of the donor tooth sources, number of roots, history of root canal treatment, and duration of tooth absence were 1.097, 1.176, 1.331, and 1.532, respectively (Aoyama et al., 2012). In this study, the training data set (i.e., the Protocol group) was analyzed in the same manner as in our previous study. The hazard function of this analysis was modeled as *h*(*t*) = *h*_0_(*t*) exp (PI), where *h*_0_(*t*) is the baseline hazard and PI is the product of each β coefficient × variable (0 or 1) (Royston & Altman, 2013). The PI was calculated as follows,

PI=1.097×source of donor tooth(0:mandibular,1:maxillary)+1.176×number of roots(0:single,1:multiple)+1.331×history of root canal treatment(0:no,1:yes)+1.532×duration of tooth absence(0:<2.5months,1:≥2.5months)

To validate our Cox model, first, the PI was calculated for all transplanted teeth in the Protocol group as the training data set. Since each factor was a binary variable, there were two patterns for each of the four factors; therefore, the PI had 2 × 2 × 2 ×2 = 16 different patterns. Sixteen patterns of PI were constructed from the combination of the four prognostic factors using the above formula. The cutoff value was set to the median of PI. All transplanted teeth were divided into two groups by the median of PI: the low‐ and high‐risk teeth. Survival curves were calculated for the low‐ and high‐risk groups using the Kaplan–Meier method. Statistical significance of the differences between the survival curves of the two groups was tested using the log‐rank test. Subsequently, the PI was calculated for all transplanted teeth in the Pre‐protocol group as the first validation data set. All transplanted teeth in the Pre‐protocol group were divided into low‐and high‐risk teeth by the median of PI. Survival curves were calculated for the low‐ and high‐risk groups and statistical significance of the differences between the survival curves of the two groups were tested. Finally, in the Post‐protocol group as the second validation data set, PI and the median PI for all transplanted teeth in the Post‐protocol group were also calculated and the survival curves of low‐and high‐risk groups were tested.

Descriptive statistics were calculated, and statistical analyses were performed using R and IBM SPSS Statistics for Windows, Version 21.0 (IBM Corp.). A *p* value of <.05 was considered statistically significant.

## 4 RESULTS

### 4.1 Patient characteristics

Table 1 shows the characteristics of tooth autotransplantation cases in the three groups. In the Protocol group, 259 autotransplanted teeth from 155 females (59.8%) and 104 males (40.2%) were included. The median ([25%, 75%]) age of the patients at the time of surgery was 40.0 ([29.0, 49.0]) years, with a range of 12–73 years (Aoyama et al., 2012). The Pre‐protocol group consisted of 95 autotransplanted teeth from 66 females (69.5%) and 29 males (30.5%). The median ([25%, 75%]) age of the patients at the time of surgery was 33.0 ([25.0, 44.0]) years, with a range of 10–69 years. The Post‐protocol group consisted of 61 autotransplanted teeth from 38 females (62.3%) and 23 males (37.7%). The median ([25%, 75%]) age of the patients at the time of surgery was 38.0 ([29.0, 49.0]) years, with a range of 12–69 years. The characteristics of donor teeth and recipient sites and others of the three groups are shown in Table 1. Table 2 shows the distribution of transplanted teeth in terms of donor teeth and recipient sites in the three groups.

**Table 2:** Distribution of transplanted teeth in terms of donor teeth and recipient sites in the Protocol, Pre‐, and Post‐protocol groups.

Protocol group			*Donor teeth*										
			*maxilla*					*mandible*					
			*n = 112(43.2)%*					*n = 147(56.8)%*					
			*Incisors*	*Premolars*		*molars*		*Premolars*		*molars*			
	*Recipient site*		*lateral*	*First*	*Second*	*Second*	*Third*	*First*	*Second*	*Second*	*Third*	*sum*	*%*
maxilla	Incisors	Central	1					3	2			6	2.3
*n* = 58	Premolars	First		1			1					2	0.8
(22.4%)		Second		1			2	3	1			7	2.7
	molars	First					14	1		1	3	19	7.3
		Second					12	3			9	24	9.3
mandible	Premolars	First	1	2				1	1			5	1.9
n=201		Second		5	1		5	3			3	17	6.6
(77.6%)	molars	First		2	1	5	28	2	1	1	44	84	32.4
		Second		1		1	26	1	1		63	93	35.9
		Third					2					2	0.8
Sum			2	12	2	6	90	17	6	2	122	259	
%			0.8	4.6	0.8	2.3	34.7	6.6	2.3	0.8	47.1		

**Pre‐protocol group**			*Donor teeth*													
			*maxilla*						*mandible*							
			*n = 41(43.2)%*						*n = 54(56.8)%*							
			*Incisors*	*Premolars*		*molars*			*Incisors*		*Premolars*		*molars*			
	*Recipient site*		*lateral*	*First*	*Second*	*First*	*Second*	*Third*	*Central*	*lateral*	*First*	*Second*	*Second*	*Third*	*sum*	*%*
maxilla	Incisors	Central			1						1	1			3	3.2
n=23		lateral							1	1					2	2.1
(24.2%)	Canine				1			1							2	2.1
	Premolars	First									2				2	2.1
		Second	1					1			1			1	4	4.2
	molars	First						2						4	6	6.3
		Second		1				1			1			2	5	5.3
mandible	Premolars	Second		1		1		2							4	4.2
*n* = 72	molars	First		2			2	10		1	1	1		10	27	28.4
(75.8%)		Second		2	1		1	11				1	1	23	40	42.1
Sum		Sum	1	6	3	1	3	28	1	2	6	3	1	40	95	
(%)		%	1.1	6.3	3.2	1.1	3.2	29.5	1.1	2.1	6.3	3.2	1.1	42.1		

**Post‐protocol group**			*Donor teeth*								
			*maxilla*				*mandible*				
			*n = 26(42.6)%*				*n = 35(57.4)%*				
			*Incisors*	*Premolars*		*molars*	*Premolars*		*molars*		
	*Recipient site*		*lateral*	*First*	*Second*	*Third*	*First*	*Second*	*Third*	*sum*	*%*
maxilla	Incisors	Central					1			1	1.6
*n* = 15		lateral	1							1	1.6
(24.6%)	Premolars	First						1		1	1.6
	molars	First		1		1				2	3.3
		Second				5	1		4	10	16.4
mandible	Premolars	Second		1	1	3			1	6	9.8
*n* = 46	molars	First				6			9	15	24.6
(75.4%)		Second				7			18	25	41
Sum			1	2	1	22	2	1	32	61	
(%)			1.6	3.3	1.6	36.1	3.3	1.6	52.5		

### 4.2 Correlation matrix for factors in each of the three groups and comparison among the three groups

**Figure 1:**
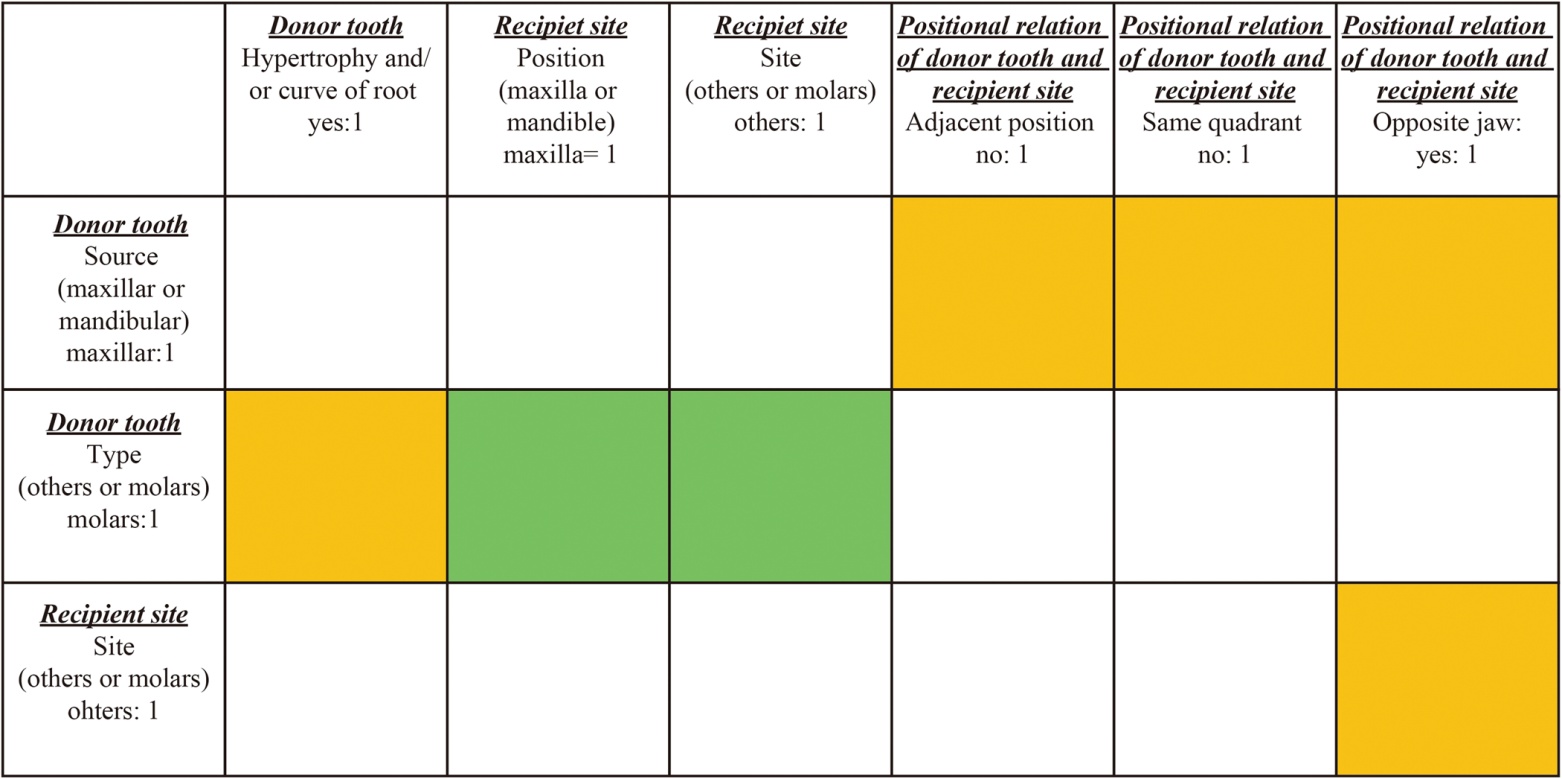
Correlation matrix for factors in each of the three groups and comparison among the three groups. The vertical and horizontal squares correspond to the variable factors listed in Table 1. In the comparison of the three groups, the seven factor combinations that were correlated were common (five positive correlations and two negative correlations). The orange and green cells indicate positive and negative correlations, respectively. “1” for each factor indicates the method of binarization. The orange box where the second row from the top intersects the leftmost column indicates a positive correlation between cases with a molar donor tooth and cases with a hypertrophy and/or curve of the root. The green box to the right of this orange box indicates a negative correlation between cases with a molar donor tooth and cases of tooth transplantation to the maxilla. Positive correlations were observed in four other combinations of factors; maxillary donor tooth and transplantation in nonadjacent positions, maxillary donor tooth and transplantation in nonopposite quadrant, maxillary donor tooth and transplantation in the opposite jaw, transplantation to the nonmolar sites and transplantation in the opposite jaw. Negative correlations were observed for combinations of molar donor teeth and transplants to nonmolar sites.

In the correlation matrix of the Protocol, Pre‐, and Post‐protocol groups, correlations were observed among 70, 35, and 18 combinations of variable factors, respectively. In the comparison of the three groups, the seven combinations of variable factors that were correlated were common: five positive correlations and two negative correlations. The orange and green cells indicate positive and negative correlations, respectively (Figure 1). In Figure 1, “1” for each factor indicates the method of binarization. For example, if there is a molar donor tooth, its factor is defined as “1” as shown in the top of the extreme left. If the root of the donor tooth has hypertrophy and/or curve of root morphology, the factor is defined as “1” as shown in the upper leftmost row. Hence, the orange box where the second row from the top intersects the leftmost column indicates a positive correlation between cases with a molar donor tooth and cases with a hypertrophy and/or curve of the root. The green box to the right of this orange box indicates a negative correlation between cases with a molar donor tooth and cases of tooth transplantation to the maxilla (Figure 1). For instance, the upper molars in the Protocol group were transplanted in the mandible in 67 patients and in the maxilla in 29 patients. In contrast, the lower molars in 111 and 13 patients were transplanted in the mandible and maxilla, respectively. The other two groups showed similar trends (Table 2). Positive correlations were observed in four other combinations of factors: maxillary donor tooth and transplantation in nonadjacent positions, nonopposite quadrant, and opposite jaw and transplantation to the nonmolar sites and transplantation in the opposite jaw (Figure 1). For example, in the Protocol group, 80 maxillary teeth were transplanted to the mandible, significantly more than the 32 teeth transplanted to the maxilla. When the recipient sites were the maxillary nonmolar sites, the donor teeth were nine mandibular teeth (six for maxillary donor teeth), and when the recipient sites were the mandibular nonmolar sites, the maxillary teeth were 14 (eight for mandibular donor teeth). The other two groups showed similar trends (Table 2). Negative correlations were observed for combinations of molar donor teeth and transplants to nonmolar sites (Figure 1). For example, in the Protocol group, three maxillary molar donor teeth were transplanted to maxillary nonmolar sites (26 transplants to molar sites) and five maxillary molar donor teeth were transplanted to mandibular nonmolar sites (62 transplants to molar sites). No mandibular molar donor tooth was transplanted to maxillary nonmolar site (13 transplants to molar sites) and three mandibular molar donor teeth were transplanted to mandibular nonmolar sites (108 transplants to molar sites). The other two groups showed similar trends (Table 2). Six of the seven correlating factors common to the three groups showed a relationship between the source (maxillary or mandible) or type (other teeth or molars) of the transplanted tooth and the recipient site.

### 4.3 Clinical outcomes

Among the 259 transplanted teeth surveyed in the Protocol group, 27 teeth (10.4%) were judged as unsuccessful cases and were considered uncensored in the statistical analysis. The remaining 232 teeth (89.6%) were considered censored during the follow‐up. The mean follow‐up time was 35.6 months, and the median ([25%, 75%]) was 28.7 months ([9.8, 58.1]), ranging from 1.8 to 101.2 months. In this group, 23 of the 27 unsuccessful transplanted teeth in the Protocol group were either extracted or avulsed during the observation period. The mean and median ([25%, 75%]) follow‐up time was 35.6 and 28.7 months ([9.8, 58.1]), (range: 1.8–101.2); 23 of the 27 unsuccessful transplanted teeth in the Protocol group were either extracted or avulsed during the observation period for better flow and readability. The causes were failure of peritransplant tissue healing (17 teeth, 63.0%) and root resorption (10 teeth, 37.0%) (Aoyama et al., 2012). In the Pre‐protocol group, among the 95 transplanted teeth surveyed, 17 (17.9%) were judged as unsuccessful cases and were considered uncensored in the statistical analysis. The remaining 78 teeth (82.1%) were considered censored during the follow‐up. The mean follow‐up time was 40.2 months, and the median ([25%, 75%]) was 24 months ([9.4, 65.6]), ranging from 0.3 to 145.7 months. In the Pre‐protocol group, 17 teeth were extracted or fell out during the observation period. The median period from transplantation to extraction was 29.6 months, ranging from 0.3 to 112.3 months. The causes were failure of peritransplant tissue healing (12 teeth, 70.6%) and root resorption (five teeth, 29.4%). In the Post‐protocol group, among the 61 transplanted teeth surveyed, two teeth (3.3%) were judged as unsuccessful cases and were considered uncensored in the statistical analysis. The remaining 59 teeth (96.7%) were considered censored during the follow‐up. The mean follow‐up time was 22.5 months, and the median ([25%, 75%]) was 22.1 months ([9.0, 35.4]), ranging from 2.1 to 52.7 months. Two teeth were extracted during the observational period. The periods from transplantation to extraction was 8.2 and 9.9 months (median: 9.1 months). The causes were failures of peritransplant tissue healing.

### 4.4 Overall life table

**Figure 2:**
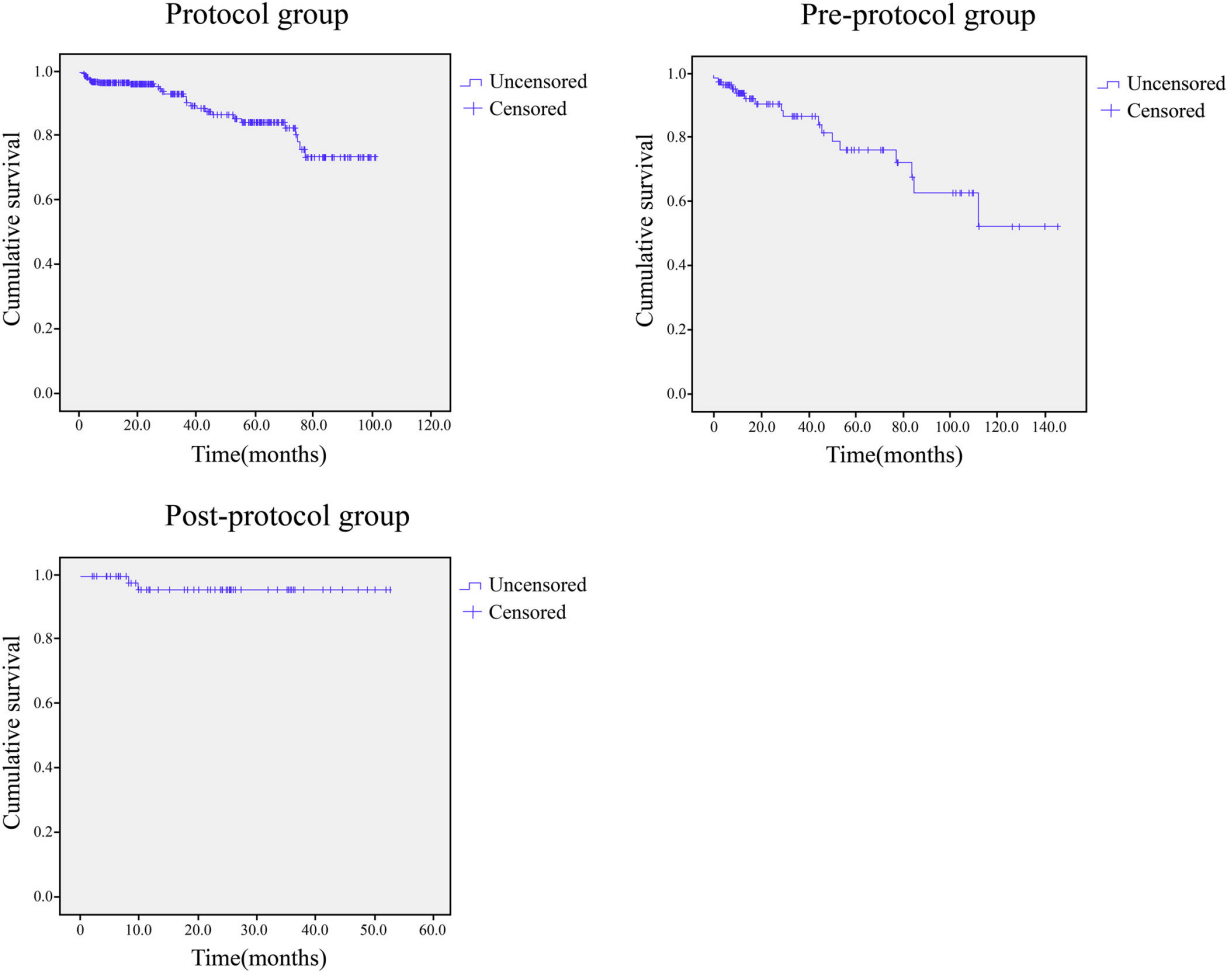
The overall life tables for the autotransplanted teeth in the Protocol, Pre‐protocol, and Post‐protocol groups. The overall life tables for autotransplanted teeth in the three groups were analyzed using the Kaplan–Meier method. A significant improvement in the cumulative survival rates was observed in the Protocol and Post‐protocol groups.

The overall 1‐year survival rate using the Kaplan–Meier method and the 5‐year cumulative survival rate in the Protocol and Pre‐protocol groups were 96.8% and 94.2% and 84.4% and 76.5%, respectively (Aoyama et al., 2012). In the Post‐protocol group, the overall 1‐year survival rate using the Kaplan–Meier method was 95.8%, and the 4‐year cumulative survival rate was 95.8%. (Figure 2).

### 4.5 Validation using the PI

**Figure 3:**
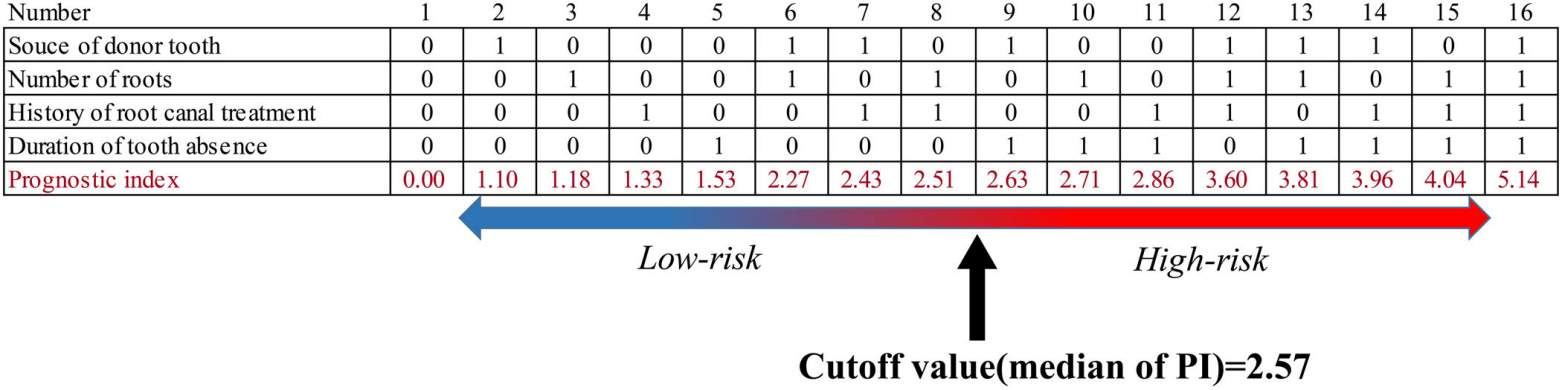
Sixteen patterns of PI and tooth autotransplantations divided into low‐ and high‐risk groups. Sixteen patterns of PI were constructed from a combination of four prognostic factors, and the results of all tooth autotransplantations were divided into low‐ and high‐risk groups according to the median of PI (2.57). PI, prognostic index.

The results of the multivariate Cox proportional hazards regression analysis with a stepwise procedure of the Protocol group analyzed again as a training data set were consistent with those previously reported (Aoyama et al., 2012). Sixteen patterns of PI were constructed using the previously described formula and the cutoff value was set to the median of PI (2.57). The outcomes of all the autotransplanted teeth were divided into low‐ and high‐risk groups according to the median PI (Figure 3).

**Figure 4:**
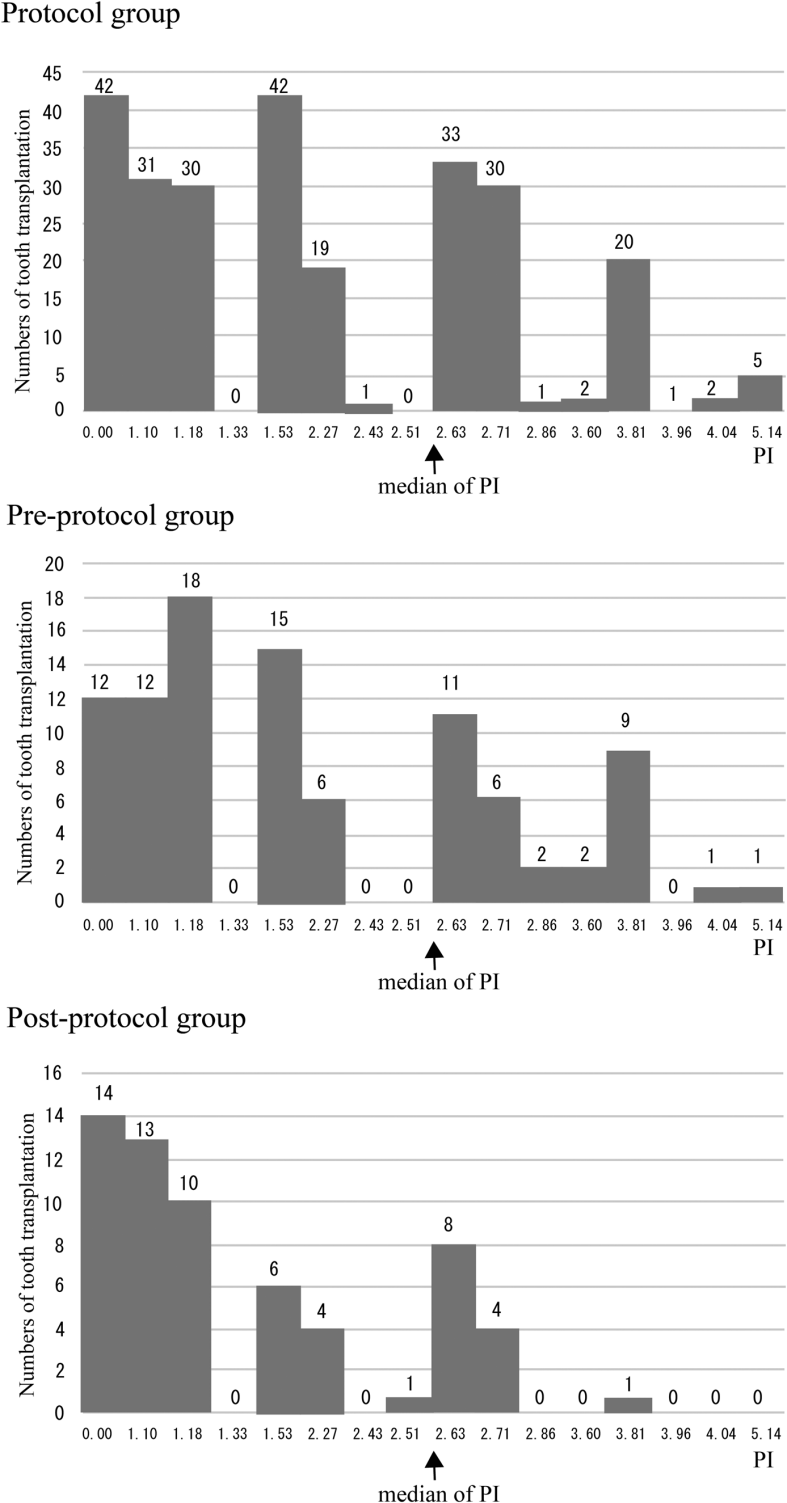
Histogram of the PI of the three groups. The median of PI is 2.57. In the Protocol group, 165 (63.7%) and 94 (36.3%) autotransplantations were considered as low‐risk and high‐risk, respectively. In the Pre‐protocol group, the low‐risk group comprised 63 (66.3%) autotransplantations, with 32 (33.7%) considered as high‐risk. In the Post‐protocol group, 48 autotransplantations (78.7%) were categorized as low‐risk and 13 (21.3%) as high‐risk. PI, prognostic index.

In the Protocol, Pre‐protocol, and Post‐protocol groups, 165 (63.7%), 63 (66.3%), and 48 (78.7%) autotransplanted teeth were classified as low‐risk, respectively, while 94 (36.3%), 32 (33.7%), and 13 (21.3%) were classified as high‐risk, respectively (Figure 4).

**Figure 5:**
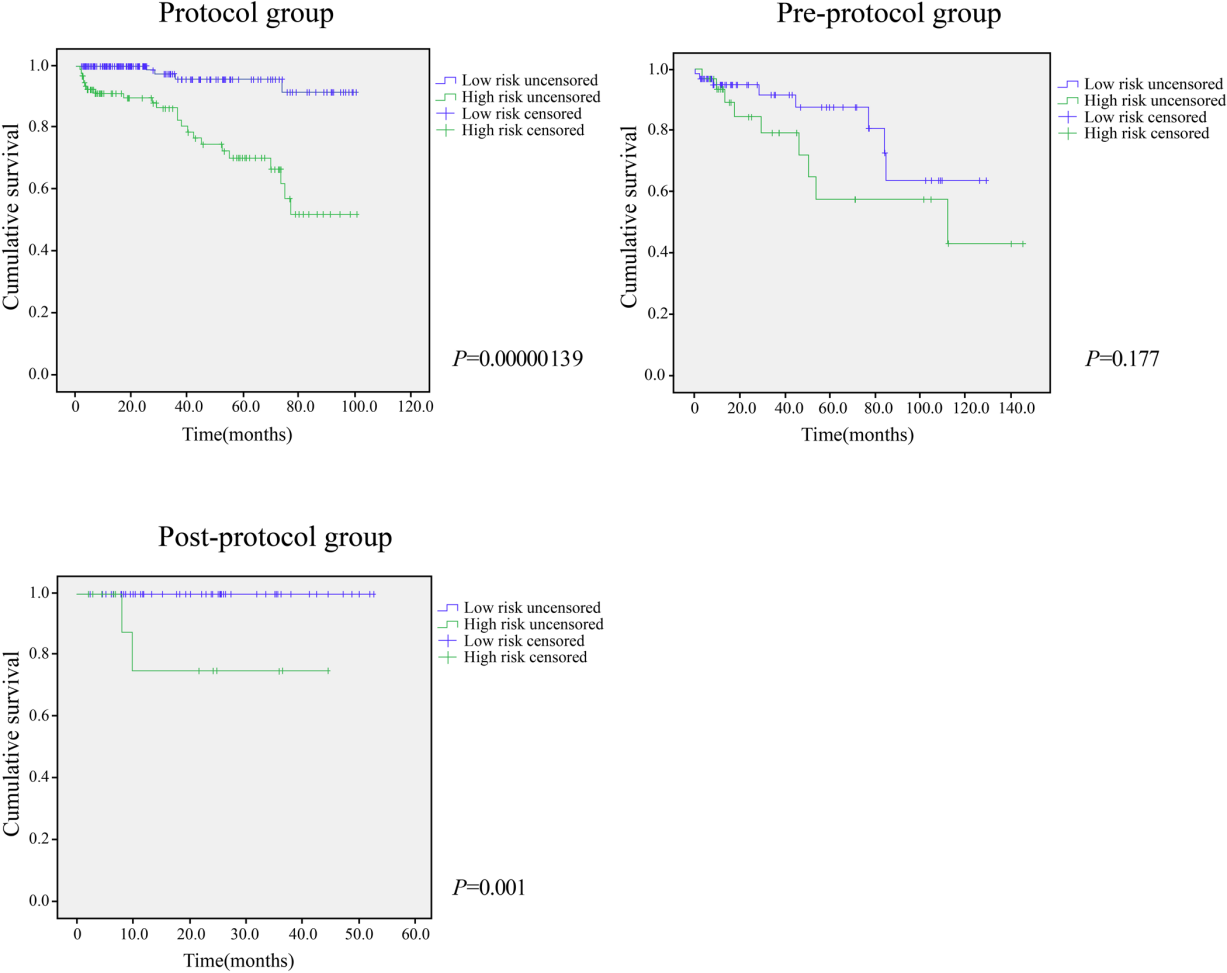
Life tables for low‐ and high‐risk autotransplanted teeth were divided according to the median of PI. In the Protocol group, the *p* value by log‐rank test was significant and was therefore used as a test set to calculate the PI with the Cox proportional hazard model. In the Pre‐protocol group, although the *p* value by log‐rank test was not significant, the cumulative survival rate curves of the low‐ and high‐risk groups were separated, and the low‐risk survival rate was better. In the Post‐protocol group, the *p* value by log‐rank test was highly significant. PI, prognostic index.

The life tables for low‐ and high‐risk autotransplanted teeth were divided according to the median of PI. In the Protocol group, the cumulative survival rate curves of the low‐ and high‐risk groups diverged significantly, and the *p* value, as calculated with the log‐rank test was significant (*p* = .000000139). This data set was used as a training data set to calculate the PI with the Cox proportional hazards model. In the Pre‐protocol group, although the *p*‐value by the log‐rank test was not significant (*p* = .177), the cumulative survival rate curves of the low‐ and high‐risk groups were separated. The low‐risk survival rate was also higher. In the Post‐protocol group, the *p*‐value obtained by the log‐rank test was highly significant (*p* = .001). The cumulative survival rate curves of the low‐ and high‐risk groups also showed significant differences (Figure 5).

## 5 DISCUSSION

In our series of studies regarding the prognostic factors of autotransplantation of teeth with complete root formation, the following four variables were statistically associated with unsuccessful transplantation of teeth with complete root formation: history of root canal treatment, number of roots, source of donor tooth, and duration of tooth absence (Aoyama et al., 2012). Several studies have reported the same statistical analysis method of tooth autotransplantation as that in this study (Jang et al., 2016; Mejàre et al., 2004; Nagori et al., 2014; Schwartz et al., 1985; Yang et al., 2019; Yoshino et al., 2012). Notably, logistic and Cox proportional hazards models are now widely used for routine statistical analyses in oncology and other medical fields to investigate the treatment outcomes associated with patient and disease characteristics; however, such a model has no clinical value unless it can successfully predict outcomes. An indispensable aspect of prediction is to consider whether a model derived from the analysis of an original data set is transferable to similar patients at another location and whether the concept can be validated and generalized. External validation refers to the performance assessment of an established model when applied to an independent data set (Royston & Altman, 2013). Therefore, for external validation, two validation datasets were selected based on the autotransplantation of teeth, that is, the Pre‐ and Post‐protocol groups, as compared with the Protocol group. The baseline hazard function is a vital component of the Cox model; however, it has not yet been estimated. Therefore, considering the Cox model to consist of its regression coefficients and covariance matrix was reasonable. For practical applications, the main product of the Cox model is PI, which is determined by the linear predictor from the Cox model. The linear predictor is a weighted sum of the variables in the model, where the weights are the regression coefficients. In the usual situation, in which the event of interest is an adverse outcome, higher values indicate poorer prognoses (Royston & Altman, 2013; Katsuya et al., 2012; Pflug et al., 2014). Therefore, we validated our prognostic model for the autotransplantation of teeth with complete root formation based on these statistical analyses.

First, the epidemiological characteristics of root formed tooth autotransplantation cases were analyzed using a correlation coefficient matrix for the three groups. The correlation between the prognostic factors in each group was analyzed and subsequently compared among the three groups. A total of seven combinations of factors were common among the three groups, with five positive and two negative correlations. Six of the combinations were in relation to donor teeth and recipient sites. Maxillary donor teeth tended to be transplanted to the mandible, molar donor teeth tended to be transplanted to the mandibular molar area. The recipient sites other than molars were transplanted from teeth of the opposite jaw. Notably, donor maxillary tooth is one of the four prognostic factors in the Cox model. In all three groups, the recipient site tended to be the mandibular molar, regardless of whether the donor tooth was a lower or upper molar. Considering the difficulty in preparing the recipient socket, the maxillary tooth was selected as a prognostic factor (Aoyama et al., 2012). This is mainly owing to the morphological incompatibility between the maxillary and mandibular molars, especially because molars often show abnormal root morphology, which showed a positive correlation in our study. The pattern of transplantation of maxillary molars to the mandible tended to be more common in all three groups. However, the indications for transplantation of maxillary molars, specifically the third molars, should be adequately determined before transplantation to the mandible. This study is probably the first attempt to statistically analyze the epidemiological characteristics of root formed tooth autotransplantation cases.

Regarding the clinical outcomes of the three groups in this study, the treatment of 27 (10.4%) teeth in the Protocol group was considered unsuccessful. In contrast, the treatments of 17 (17.9%) and two (3.3%) teeth in the Pre‐ and Post‐protocol groups, respectively, were considered unsuccessful. The survival rate in the Pre‐protocol group was the lowest among the three groups, although the rates of all the three groups were within the ranges reported in previous studies (Almpani et al., 2015). The criteria used to designate a case as unsuccessful were failure of peritransplant tissue healing and root resorption in the three groups.

In the Protocol and Pre‐protocol groups, the 1‐year overall survival rates using the Kaplan–Meier method were 96.8% and 94.2%, respectively, and the 5‐year cumulative survival rates were 84.4% and 76.5%, respectively. Cumulative survival was better in the protocol group than in the Pre‐protocol group. In the Post‐protocol group, the 1‐year overall survival rate was 95.8%, and the 4‐year cumulative survival rate was 95.8%. The 4‐year survival rate was better than the 5‐year survival rate in the Protocol and Pre‐protocol groups. This may be partly due to the shorter observation period than in the other groups, and also because the number of participating oral surgeons was very small compared to the other two groups, which may have allowed for better selection of indicated cases, refinement of surgical techniques, and information sharing on prognostic factors.

To evaluate the validity and the reliability of our Cox prognostic model for autotransplantation of teeth with complete root formation, 16 patterns of PI were constructed from the combination of four prognostic factors selected as a Cox model. The outcomes of all tooth autotransplantations were divided into low‐ and high‐risk groups using the median of PI. First, in the Protocol group as the training data set, 165 (63.7%) autotransplantation cases were classified as low‐risk, while 94 (36.3%) were classified as high‐risk, and the cumulative survival rate curves of the low‐ and high‐risk groups diverged significantly. Subsequently, in the Pre‐protocol group as a first validation data set, and in the Post‐protocol group as a second validation data set, 63 (66.3%) and 48 (78.7%) autotransplantation cases were classified as low‐risk, while 32 (33.7%) and 13 (21.3%) were classified as high‐risk, respectively. In the Pre‐protocol group, the cumulative survival rate curves of the low‐ and high‐risk groups were separated, and the low‐risk survival rate was improved. In the Post‐protocol group, the cumulative survival rate curves of the low‐ and high‐risk groups also showed significant differences. These results demonstrate the validity of our Cox prognostic model using an external validation study with PI. More autotransplantation cases in the low‐risk group were identified in the Post‐protocol group than in the Pre‐protocol and Protocol groups. However, although the three groups were independent datasets in terms of treatment duration, seven participating oral surgeons were present in the Post‐protocol group, fewer than in the other two groups, which may have allowed oral surgeons to share prognostic information with each other.

In conclusion, our Cox proportional hazards prognostic model for autotransplantation of teeth with complete root formation is useful for predicting the prognosis by external validation using PI. This is the first validation study of the Cox prognostic model for autotransplantation of teeth with complete root formation. In future, a multicenter study is required to develop a more precise prognostic model for autotransplantation of teeth with complete root formation.

## AUTHOR CONTRIBUTIONS

**Toshiya Yoshino**: Investigation; writing‐original draft; software. **Michiko Yoshizawa**: Conceptualization; investigation; data curation; methodology; writing‐review &editing, & editing; project administration. **Shoko Aoyama**: Data curation; investigation; methodology. **Toshiko Sugai‐Toyama**: Data curation; investigation; methodology. **Niimi Kanae**: Data curation; investigation; methodology. **Nobutaka Kitamura**: Conceptualization; formal analysis; validation; software; visualization. **Tadaharu Kobayashi**: Conceptualization; project administration; supervision.

## CONFLICT OF INTEREST STATEMENT

The authors declare no conflicts of interest.

## Data Availability

Raw data were generated at Niigata University. Derived data supporting the findings of this study are available from the corresponding author, Michiko Yoshizawa, on request.

## 1

Figure A1
